# The influence of stereoscopic vision on surgical performance in minimal invasive surgery—a substudy of the IDOSP-Study (Influence of 3D- vs. 4 K-Display Systems on Surgical Performance in minimal invasive surgery)

**DOI:** 10.1007/s00423-022-02608-3

**Published:** 2022-07-22

**Authors:** Caroline Gietzelt, Rabi Datta, Jana Busshoff, Thomas Bruns, Roger Wahba, Andrea Hedergott

**Affiliations:** 1 Department of Ophthalmology, Faculty of Medicine and University Hospital Cologne, University of Cologne, Joseph-Stelzmann-Straße 9, 50931 Cologne, Germany; 2 Department of General, Visceral, Cancer and Transplantation Surgery, Faculty of Medicine and University Hospital Cologne, University of Cologne, Cologne, Germany; 3 Department of Obstetrics and Gynecology, Faculty of Medicine and University Hospital Cologne, University of Cologne, Cologne, Germany

**Keywords:** Minimal invasive surgery, Laparoscopic, 3D, 4 K, Surgical performance, Stereo vision

## Abstract

**Purpose:**

This study is a secondary analysis of the IDOSP trial published in the *Annals of Surgery* 2020. The aim of this study was to examine the influence of stereo acuity on surgical performance in a laparoscopic training parkour with 3D- versus 4 K-2D-display technique.

**Methods:**

The surgical performance of medical students (MS), non-board-certified surgeons (NBC), and board-certified surgeons (BC) was compared using 3D- versus 4 K-2D-display technique at a training parkour in a randomized cross-over trial. Stereo acuity was tested by TNO and Titmus Stereo tests.

**Results:**

Eighty-nine participants were included in this sub-trial. The median stereo acuity for all participants, measured with the Titmus test, was 25 s arc, with TNO test 30 s arc. Higher quality stereo vision, measured with the Titmus test, correlated significantly with a reduced parkour time (*r* = 0.26, *p* = 0.02) and error (*r* = 0.21, *p* = 0.048) with the 3D screen. The TNO test did not correlate significantly with parkour performance. There was no statistically significant correlation between parkour time nor error and stereo acuity using the 4 K system (*p* > 0.457 respectively). Higher age showed a significant correlation with lower stereo acuity measured with TNO (*r* = 0.21, *p* = 0.014), but not with the Titmus test (*r* =  − 0.7, *p* = 0.39). Seven percent of the group “NBC and BC” showed reduced stereo acuity > 120 s arc with the Titmus test and 3% with the TNO test.

**Conclusion:**

High-quality stereo vision is of utmost importance for surgical skills using a 3D-display system. This was most obvious for MS and for tasks that place particularly high demands on hand–eye coordination. The Titmus test was more precise than the TNO test to predict the benefit of a 3D monitor system. Experience and fine motor skills could partly compensate for a poorer stereo acuity.

**Trial registration:**

This trial was registered at clinicaltrials.gov (trial number: NCT03445429, registered February 26, 2018).

## Introduction

High-quality stereopsis offers an advantage when performing fine motor tasks under direct view [1]. However, stereopsis does not appear advantageous to task performance under 2D viewing conditions, such as in video-assisted surgery [2], which might be one of many reasons why some complications are more frequent in 2D laparoscopic surgery than in open abdominal surgery [3].

In 3D laparoscopy, the images from two cameras are fused and presented as a single 3D image by a special monitor and viewed through polarized glasses [4]. With advancing technology, 3D was shown to improve the laparoscopic precision of novice and experienced surgeons as well as shorten the operative time [4–7]. Even though it is widely acknowledged that high-quality stereopsis is necessary for the use of 3D laparoscopic imaging systems in surgery [1, 7], to our knowledge there has not been a study correlating the effect of the measured stereo acuity on the performance in 3D laparoscopy and 4 K-2D laparoscopy before.

There are several methods of testing stereo vision [8]. In all of them, the principle is similar. Slightly different images are displayed for each eye so that people with existing stereo vision will perceive a 3D image. In everyday clinical practice, the following tests are mainly used to test stereopsis: random dot stereo tests and contour stereo tests. In random dot stereo tests, images of stereo figures are embedded in a background of random points (Lang test, TNO test) [9]. In contour stereo tests, images are separated horizontally to present figures to each eye (Titmus test) [10]. Contour stereo tests in contrast to random dot stereo tests have the disadvantage of monocular cues [9]. However, the Titmus test, which is a contour test, has been shown to be more sensitive to small changes in stereo acuity [11].

This study is a secondary analysis of the IDOSP trial published in the *Annals of Surgery* 2020 [12]. The primary endpoint of the IDOSP trial was a comparison of the surgical performance defined by the items “time in seconds” and “number of mistakes” between a 3D- and 4 K-display system.

The aim of this secondary analysis was to correlate stereo acuity with surgical performance in 3D laparoscopy and 4 K-2D laparoscopy. Furthermore, two different common stereo tests (TNO, Titmus) should be evaluated regarding their correlation with surgical performance in 3D laparoscopy and 4 K-2D laparoscopy. The effects of experience, fine motor skills, and age were assessed. As this is a secondary analysis and the primary endpoint of the IDOSP-Study was different from this secondary analysis, the results in this publication can therefore only be perceived as descriptive.

## Materials and methods of investigation

### Inclusion and exclusion criteria

Inclusion criteria for the IDOSP-Study [12, 13] were medical students, surgeons in training, and board-certified surgeons who gave their written informed consent and were > 18 years of age.

Medical students with any experience in laparoscopic surgery, or any candidates (medical students and surgeons) with any experience in the laparoscopic training parkour, non-correctable vision disorders, known impaired stereoscopic vision, or manual skill disorders were excluded from the study.

### Participants

Inexperienced novices (medical students), non-board-certified, and board-certified surgeons were examined by an experienced ophthalmologist (A.H., C.G.) for stereoscopic vision and exclusion of manifest strabismus with five qualitative and semi-quantitative tests: Bagolini striated glasses test (near and far distance); Lang (II), Titmus, and TNO stereo tests (near distance); and cover/uncover test (near and far distance). Details concerning the most relevant stereo tests for this study are shown in Fig. 3: Titmus test (Vision Assessment Corporation, 2007, IL, USA) and TNO test (Laméris Instrumenten, 3rd edition, Utrecht, Netherlands). Furthermore, monocular visual acuity was tested (far distance) and anterior segment and central fundus were screened for relevant anomalies.

### Instrument set-up

A passive polarizing 3D- and a 4 K-display system (2 arms) were used to perform different tasks in a minimal invasive/laparoscopic training parkour (3D laparoscopic system “Einstein Vision 2.0” (10 mm 308 camera, 3D full high-definition 3200 monitor, Aesculap AG, Tuttlingen, Germany) and 2D-4 K System “Visera 4 K Ultra High Definition” (10 mm 308 camera, 5500 monitor, Olympus Medical system Olympus Europa SE & Co. KG, Hamburg, Germany)). The position of the complete laparoscopic training parkour, the camera position in the laparoscopic training system, and the distance from study subject to the screens were standardized. The distance between the participant and the 3D screen or the 4 K screen was 130 cm.

### Tasks

Each participant had to perform two runs of the parkour with the two different display systems. The parkour consisted of 5 tasks for medical students and 5 + 2 tasks for surgeons, which were each repeated three times. The five tasks were “rope pass,” “paper cut,” “pegboard transfer,” “needle threading,” and “needle recapping.” The two additional tasks with an increased degree of difficulty were “circle cutting” and “knot tying.” The first run of the parkour was performed with a randomized display system, the second with the other one. A detailed description of the minimal invasive training parkour can be found in the published study protocol [13]. Surgical performance was measured by performance time (seconds) and the number of mistakes (error defined as any deviance from perfect performance).

### Subjective impression of the participants

The participants’ task load for each run was evaluated by the National Aeronautics and Space Administration Task Load Index (NASA-TLX).

### Ethics

This trial was approved by the Ethics Committee of the University of Cologne (No. 17–388). All subjects gave their written informed consent before randomization by an independent data trustee.

### Statistics and mathematical analysis

Statistical analysis was performed using Excel (Microsoft Excel for Mac, Version 15.29.1, Microsoft, USA) and SPSS (IBM SPSS Statistics, Version 25.0, IBM, USA). Each participant’s median overall parkour time and median overall parkour error per screen system were calculated and used for further evaluation.

Mean and standard deviation were calculated for normally distributed data. For ordinal scaled data, median and range were given. For further statistical analysis, the stereo acuity results were grouped as either “normal” (25 s arc or better, subgroup 1), “subnormal” (> 25 to 40 s arc, subgroup 2) or “moderate to reduced” stereopsis (50 s arc and worse, subgroup 3) [14]. Additionally, within subgroup 3 the number of participants with stereo acuity worse than 120 s arc, defined as “reduced,” was analyzed. This subgroup was too small for further statistical analysis.

Correlations were tested by Spearman’s rho. The threshold for statistical significance was set to *p* < 0.05. The paired Student *t*-test was used to compare means between groups for normally distributed data. For ordinal data and data without normal distribution, the Wilcoxon rank-sum test was used.

## Results

### Participants

Between February and October 2019, a total of 89 participants of the IDOSP-Study completed stereo acuity tests as well as the training parkour and were included in this sub-trial. Forty-seven were medical students (MS, mean age 24.5 $\pm$ 4.1 years; 27 male, 20 female), 16 non-board-certified surgeons (NBC, mean age 30.5 $\pm$ 5.5 years; 4 male, 12 female), and 26 board-certified surgeons (BC, mean age 42.5 $\pm$ 7.4; 24 male, 2 female). Epidemiologic data can be seen in Table 1.

**Table 1:** Epidemiologic data

	MS (*n* = 47)	NBC (*n* = 16)	BC (*n* = 26)
Sex, *n* (%)			
Male	27 (57%)	4 (25%)	24 (92%)
Female	20 (43%)	12 (75%)	2 (8%)
Age (years)			
Mean ± SD	24.5 ± 4.1	30.5 ± 5.5	42.5 ± 7.4
Median	23	29	41
Range	20 to 41	26 to 47	32 to 63
Titmus test			
Median	25	25	25
Range	25 to 200	25 to 3550	25 to 160
Distribution of Titmus subgroups (%)	MS (*n* = 47)	NBC (*n* = 16)	BC (*n* = 26)
Subgroup 1 (≤ 25 s arc)	70% (*n* = 33)	56% (*n* = 9)	81% (*n* = 21)
Subgroup 2 (> 25 to 40 s arc)	19% (*n* = 9)	31% (*n* = 5)	8% (*n* = 2)
Subgroup 3 (≥ 50 s arc)	11% (*n* = 5)	13% (*n* = 2)	12% (*n* = 3)
TNO test			
Median	30	60	30
Range	15 to 120	15 to 120	15 to 240
Distribution of TNO subgroups (%)	MS (*n* = 47)	NBC (*n* = 16)	BC (*n* = 24)
Subgroup 1 (≤ 25 s arc)	28% (*n* = 13)	6% (*n* = 1)	21% (*n* = 5)
Subgroup 2 (> 25 to 40 s arc)	55% (*n* = 26)	38% (*n* = 6)	38% (*n* = 9)
Subgroup 3 (≥ 50 s arc)	17% (*n* = 8)	56% (*n* = 9)	42% (*n* = 10)

Manifest strabismus was excluded by cover and uncover test for far and near fixation. Visual acuity was at least 0.6 (decimal scale) in all eyes; 88% of right eyes and 91% of left eyes had a visual acuity of 1.0 or better.

The Bagolini test was positive for far and near fixation in all subjects. The Lang II test was positive in 85 participants (200 s arc), whereas four had reduced stereovision (400 s arc: 2 participants, 600 s arc: 2 participants).

The median stereo acuity for all participants, measured with the Titmus test, was 25 s arc (25 to 3550 s arc), with TNO test 30 s arc (15 to 240 s arc). The median stereo acuity for the subgroups MS, NBC, and BC can be seen in Table 1. The stereo acuity of the subgroup MS was significantly higher compared to the subgroups NBC and BC (MS vs BC *p* = 0.024, MS vs NBC *p* = 0.002).

There was a statistically significant correlation between the results of the TNO and Titmus test (*r* = 0.34, *p* = 0.001). When assigned to subgroups, only 22% of participants showed full stereo acuity (25 s arc or better) with TNO compared to 71% with the Titmus test. Forty-seven percent showed subnormal stereo acuity (> 25 to 40 s arc) with TNO compared to 18% with the Titmus test. Thirty-one percent showed reduced stereo acuity (50 s arc or worse) with TNO compared to 11% with the Titmus test (Table 2). Seven percent of NBC and BC showed reduced stereo acuity > 120 s arc with the Titmus test and 3% with the TNO test. Sixty percent of all participants showed better stereo acuity levels when tested with the Titmus test compared to the TNO test. The distribution of stereo acuity for the Titmus and TNO test for MS, NBC, and BC is presented in Table 1.

**Table 2:** Overall distribution of stereo acuity comparing the Titmus and TNO test

s arc	Titmus test	TNO test
≤ 25	71%	22%
> 25 to 40	18%	47%
≥ 50	11%	31%

Higher age showed a significant correlation with lower stereo acuity measured with TNO (*r* = 0.21, *p* = 0.014), but there was no significant correlation between age and results of the Titmus test (*r* =  − 0.7, *p* = 0.39).

There was no significant correlation between NASA-TLX and stereo acuity measured by the Titmus (*r* =  − 0.156, *p* = 0.149) or TNO test (*r* =  − 0.038, *p* = 0.727).

### Parkour time and error

Considering the 5 tasks performed by all participants, participants were significantly faster and had fewer errors when using 3D instead of 4 K-2D. This difference was statistically significant as well for the subgroup MS, but not for NBC and BC (*p* < 0.01 respectively). Table 3 details results.

**Table 3:** Result details

	MS (*n* = 47)	NBC (*n* = 16)	BC (*n* = 26)
5 tasks (mean ± SD)			
Parkour time 3D (s)	557.8 ± 195.8	717.2 ± 556.4	336.3 ± 130.2
Parkour time 4 K (s)	838.6 ± 338.4	864.0 ± 671.7	382.4 ± 109.2
*p*	< 0.01	< 0.01	< 0.01
Parkour error 3D	8.8 ± 5.7	10.5 ± 6.8	6.2 ± 3.7
Parkour error 4 K	12.7 ± 9.2	13.9 ± 10.3	6.3 ± 3.2
*p*	< 0.01	< 0.01	< 0.01
5 + 2 tasks		NBC and BC (***n*** = 42)	
Parkour time 3D (s)		849.7 ± 411.9	
Parkour time 4 K (s)		995.5 ± 512.7	
* p*		0.039	
Parkour error 3D		10.7 ± 7.4	
Parkour error 4 K		12.5 ± 10.0	
* p*		0.21	
	Titmus subgroup 1	Titmus subgroup 2	Titmus subgroup 3
5 tasks (mean ± SD)			
Parkour time 3D (s)	459.7 ± 206.5	740.4 ± 538.7	500.1 ± 213.9
Parkour time 4 K (s)	657.1 ± 336.0	1026.3 ± 688.7	546.9 ± 279.4
*p*	< 0.01	< 0.01	< 0.01
Parkour error 3D	7.7 ± 5.3	10.3 ± 7.1	9.0 ± 3.1
Parkour error 4 K	10.5 ± 8.9	13.3 ± 8.3	11.0 ± 7.5
*p*	< 0.01	< 0.01	< 0.01
	TNO subgroup 1	TNO subgroup 2	TNO subgroup 3
5 tasks (mean ± SD)			
Parkour time 3D (s)	457.3 ± 178.6	539.9 ± 282.1	556.1 ± 420.8
Parkour time 4 K (s)	647.9 ± 299.1	773.1 ± 454.4	656.7 ± 458.4
*p*	< 0.01	< 0.01	< 0.01
Parkour error 3D	8.1 ± 6.6	8.4 ± 5.4	8.7 ± 5.4
Parkour error 4 K	9.5 ± 6.1	12.6 ± 10.8	10.1 ± 6.2
*p*	< 0.01	< 0.01	< 0.01

The technical level of participants showed a significant correlation to the parkour seconds (*r* =  − 0.28, *p* = 0.011), but not to parkour error using the 3D screen (*r* =  − 0.18, *p* = 0.090).

Sixty-one percent of the participants were faster when using 3D, and 62% had fewer errors. This effect was strongest for MS (67% and 64%) and NBC (60% and 73%), whereas it was weaker for BC (52% and 54%).

When considering 5 + 2 tasks with an increased degree of difficulty for NBC and BC, participants performed significantly faster but did not have statistically significantly fewer errors when using 3D instead of 4 K-2D (*p* < 0.039 respectively). Table 3 shows detailed results.

### Parkour time and error depending on the degree of stereo acuity

**Fig. 1:**
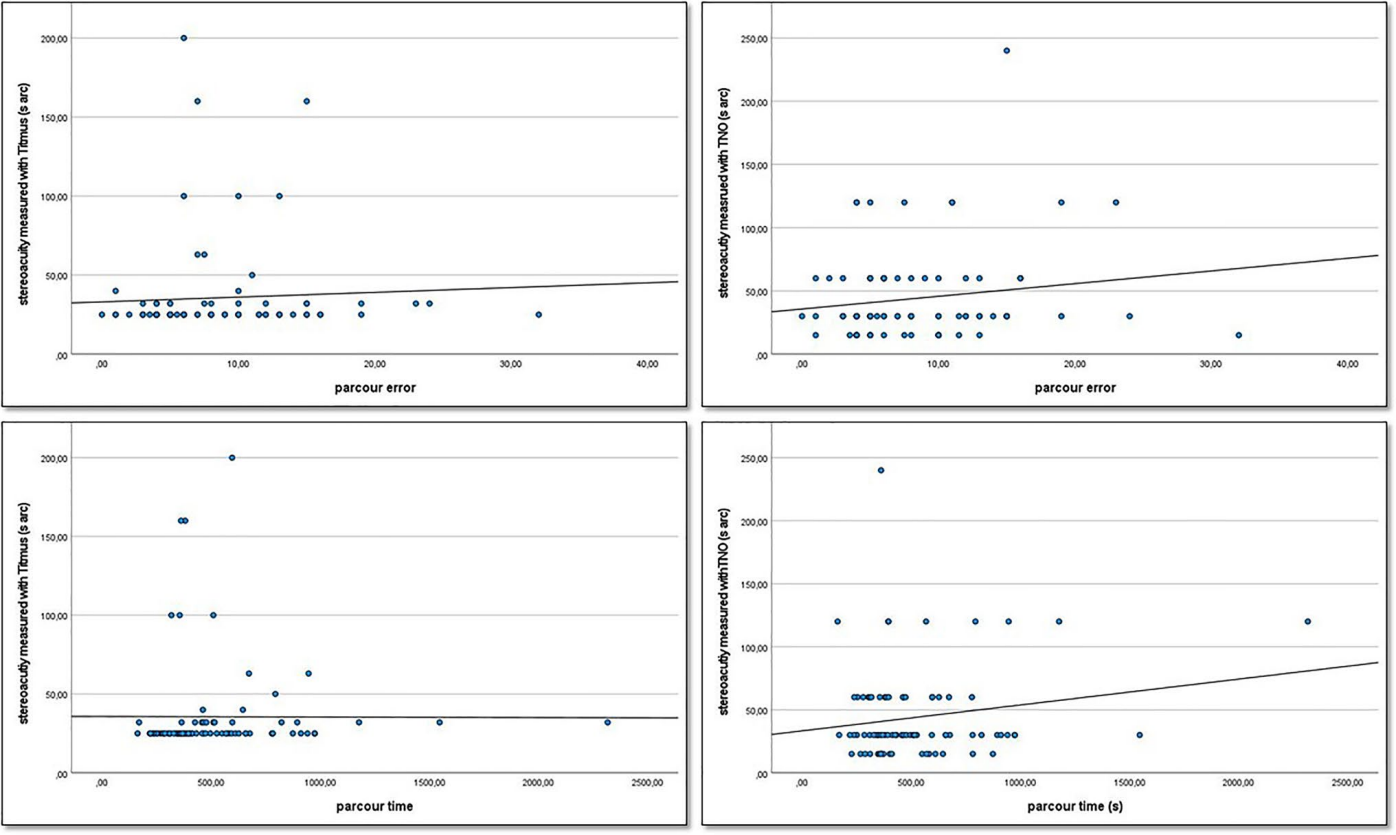
Scatter plots showing the correlation between stereo acuity measured by the Titmus or TNO test with parkour time and error

The participants’ 3D overall parkour seconds were significantly higher in Titmus subgroup 2 than in subgroup 1 (*p* = 0.002). Both the participants’ 3D overall parkour seconds (*r* = 0.26, *p* = 0.02) and the 3D overall parkour error (*r* = 0.21, *p* = 0.048) showed a statistically significant correlation with the Titmus test. Scatter plots with regression lines are shown in Fig. 1. The mean overall parkour error showed a trend to increase with decreasing TNO and the mean overall parkour time showed a trend to decrease with decreasing TNO. However, this trend was not statistically significant and there was no statistically significant correlation between 4 K overall parkour seconds or 4 K overall parkour error and stereo acuity measured by the TNO or Titmus test.

**Fig. 2:**
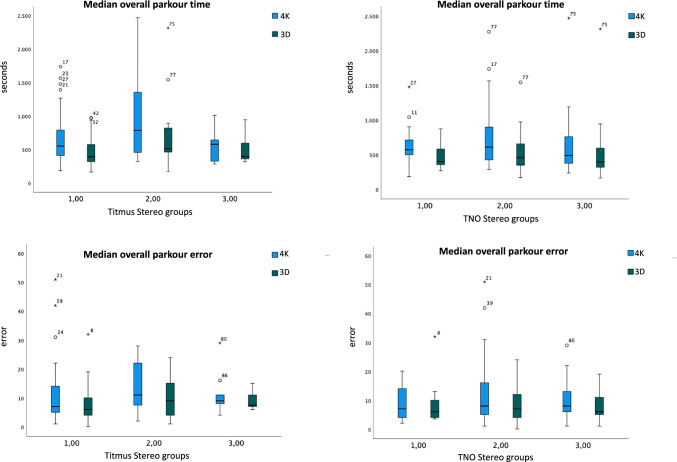
Boxplots of 4 K-2D (blue) versus 3D (green) median overall parkour time and error for three Titmus and for three TNO stereo subgroups: subgroup 1 with 25 s arc or better, subgroup 2 with > 25 to 40 s arc, and subgroup 3 with 50 s arc and worse

When assigned to stereo subgroups, in all subgroups we could confirm the results from the IDOSP-Study, where the participants’ overall parkour time was significantly faster and parkour error was significantly lower with the 3D system. This effect was statistically significant for all subgroups (*p* < 0.002 respectively) as shown in Table 3. These results can also be seen in Fig. 2.

When considering 5 + 2 tasks with an increased degree of difficulty for NBC and BC, the participants’ 3D overall parkour seconds showed a statistically significant correlation with Titmus, but not with the TNO test. The correlation coefficient *r* was *r* = 0.34 (*p* = 0.027) and therefore higher than for the overall parkour error and seconds. There was no statistically significant correlation between 3D overall parkour error, 4 K overall parkour seconds, and 4 K overall parkour error and stereo acuity measured by the TNO or Titmus test. Details are shown in Table 3.

## Discussion

Participants of the IDOSP-Study were significantly faster and had fewer errors in the surgical parkour when using 3D instead of 4 K in all subgroups of level of experience and in all subgroups of level of stereo acuity. This study showed a significant correlation between stereo vision, measured by the Titmus test, and the participant’s median 3D parkour performance (time and error). This difference was most obvious for MS and when tasks were included that placed particularly high demand on hand–eye coordination (5 + 2 tasks with an increased degree of difficulty). High-quality stereo vision of the surgeon offered no advantages or disadvantages when using the 4 K-2D-display system.

In contrast to the Titmus test, the TNO test showed the same trend but did not correlate significantly with parkour performance. One reason could be that both the Titmus test, a contour stereo test, and the 3D screen system work with polarizing glasses. In contrast, red-green glasses are used with the TNO test, a random dot stereo test. Based on the results of our study, the Titmus test is better suited to predict whether a subject will benefit from a 3D monitor system using the “state-of-the-art” passive polarizing technique.

Another reason for the difference in correlation of the two different stereo tests with parkour performance is the different nature of these two tests. As previously described in existing literature, measured stereo acuity was better when using Titmus test compared to TNO test in our study (median stereo acuity measured with the Titmus test was 25 s of arc while the median stereo acuity measured with the TNO test was 30 s of arc in our study). This underestimation of stereopsis using the TNO test has been shown by other studies before [10] and is due to various reasons.

**Fig. 3:**
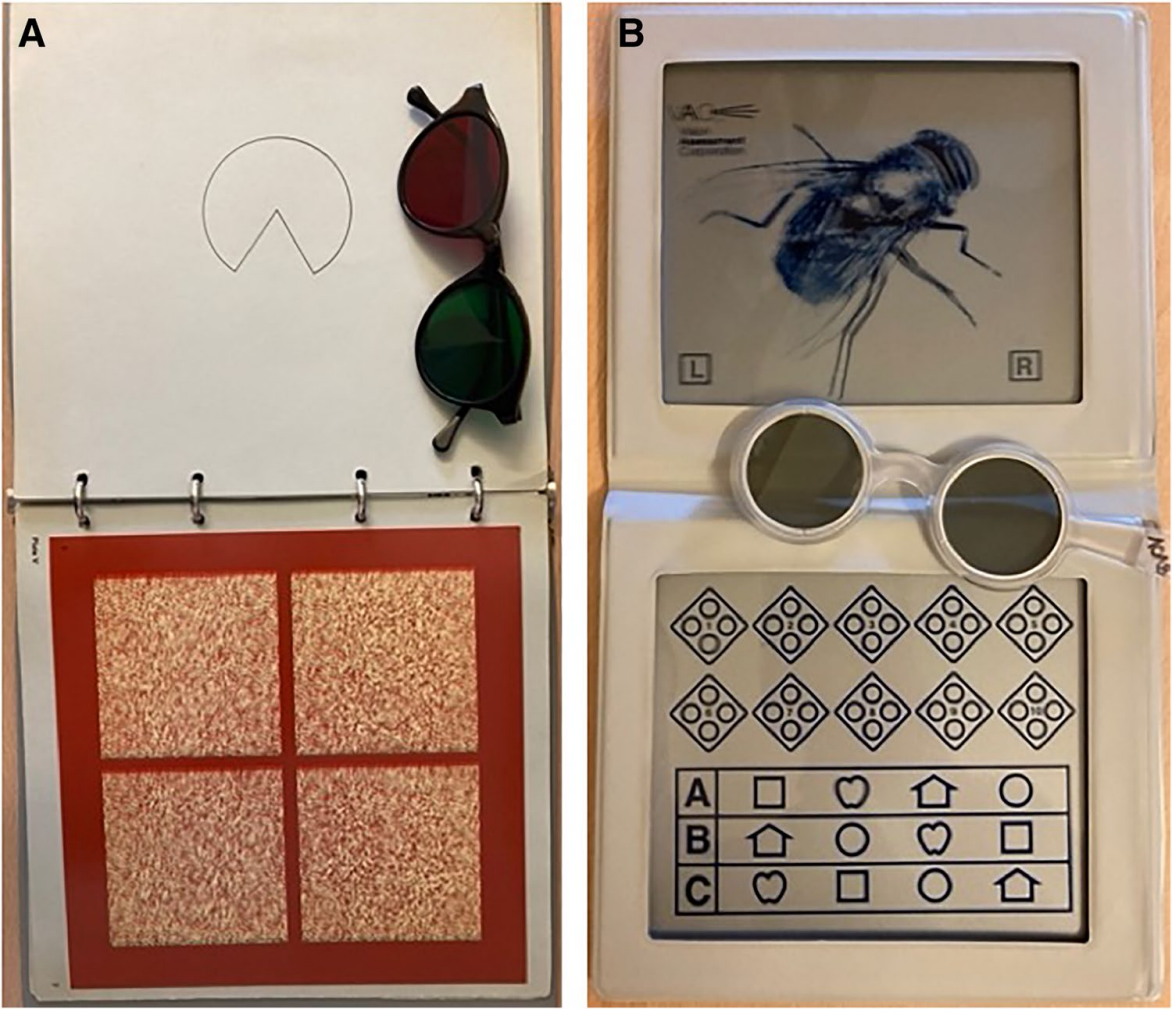
**A** The TNO stereo test uses a random dot pattern and requires anaglyph red and green glasses (worn over prescription glasses). The test contains several plates with concealed test items (disks with a sector missing) that only can be seen with binocular vision. Plates V–VII were used for the determination of stereoscopic sensitivity. Measure disparity values were 480, 240, 120, 60, 30, and 15 s of arc. **B** The Titmus stereo test is a contour stereo test; images are separated horizontally in order to present figures to each eye. This test requires cross-polarized filters (worn over prescription glasses). The second part of the test (circles) was used for the determination of stereoscopic vision. Subjects with stereo vision can see one out of four circles per object coming forward. Measure disparity values were 400, 200, 160, 100, 63, 50, 40, 32, 25, and 20 s of arc

Both tests differ in the number of items and the tested stereo sensitivity values (see Fig. 3). Measure disparity values of the TNO test are 480, 240, 120, 60, 30, and 15 s of arc (6 levels), and of the Titmus test 400, 200, 160, 100, 63, 50, 40, 32, 25, and 20 s of arc (10 levels). Therefore, the TNO test does not allow the measurement of such fine gradations in the area of excellent stereopsis as the Titmus test. In our study, both tests showed a highly significant correlation to each other (Wilcoxon rank-sum test; *p* = 0.001), but 60% of all participants showed better stereo acuity levels when tested with the Titmus test compared to the TNO test.

The TNO test, being a random dot stereo test, measures global stereopsis (or cyclopean stereopsis), which depends on disparity-selective neurons in the primary visual cortex [8, 15]. The Titmus test, being a contour stereo test, on the other hand, measures local stereopsis, which has a different neural correlate [8].

These neural processes involve a stronger stimulus for vergence of the eyes which is necessary for motor alignment and therefore depth perception [16]. Furthermore, the monocularly visible contours of the Titmus test can aid stereopsis [10].

The finding from this study correlates to evidence from existing literature, which has found the Titmus test to be more sensitive to small changes in stereovision [11].

Biddle et al. examined stereo acuity in 66 practicing surgeons across a range of surgical specialties [17]. Stereo acuity was tested with the TNO, Titmus, and Frisby test. In their study, reduced stereo acuity was defined as > 120 s arc for the TNO and Titmus test and > 250 s arc for the Frisby test. While most surgeons had high-grade stereo acuity, there were 2–14% of surgeons with reduced stereopsis and some with no stereopsis. They concluded that high-grade stereopsis seems not a universal requirement for a career in surgery. Our study confirms these results: 7% of the group “NBC and BC” showed reduced stereo acuity > 120 s arc with the Titmus test and 3% with the TNO test. However, Biddle et al. did not compare stereo acuity with surgical performance.

Comparable to our study, Fergo et al. [18] found stereo blindness in approximately 10% of 300 medical doctors in general surgery, gynecology, and urology, tested with random dot E stereo test. Prevalence increased with age. In our study, higher age showed a significant correlation with lower stereo acuity measured with TNO, but there was no significant correlation between age and results of the Titmus test. These findings are in line with Garnham et al. [19], who examined 60 normal subjects aged 17–83 years using TNO, Titmus, Frisby near, and Frisby-Davis distance stereo tests. Subjects showed some decline in stereo acuity with age by all tests. Nevertheless, a large drop in stereo acuity was reported in some older subjects using the TNO test. Garnham et al. concluded that this was probably due to difficulty overcoming the dissociative effect of the test rather than a true reduction in cortical disparity detection. Therefore, worse stereo acuity with age seems dependent on the stereo test used.

Another reason for worse stereopsis with age might be an insufficient correction of refractive error, especially presbyopia. Tuna et al. [20] investigated the impact of refractive errors on binocular visual acuity while using the Da Vinci robotic system console. The authors concluded that myopia greater than 1.75 diopters, presbyopia greater than 1.25 diopter (D), and hypermetropia regardless of grade must always be corrected for optimum performance. Presbyopia and hypermetropia had a significant effect on the 3D vision of robotic surgeons (without their complete awareness). In our study, stereo acuity was measured with the participant’s own near addition, which was also worn for the parkour performance. Some of the older surgeons might have had not sufficient near addition for the 40 cm distance necessary for the stereo test, but sufficient for the 130 cm screen distance.

Suleman et al. [21] examined 104 medical students for stereo vision (“graded circle test”) before the students performed Minimally Invasive Surgical Simulation Training sessions. They found that depth perception defects (defined as ≥ 60 s arc) compromised beginners’ ability to perform basic laparoscopic skills. Through laparoscopic simulator training, individuals with depth perception defects could improve these basic skills by a proportion comparable to that of people without defects. These findings are confirmed by the results from our study, where MS showed a greater correlation between stereo acuity and parkour performance than experienced surgeons did.

Barry et al. [22] compared the performance of participants with strabismus to that of age-matched controls in a validated surgical training module. Despite the statistical difference between participants with strabismus and controls, some individuals with poor stereopsis were able to perform quite well on a specific surgical task. Our study and previous literature show that a relevant percentage of surgeons had been able to become surgeons despite reduced stereo acuity. However, because this study is a secondary analysis of the IDOSP-Study, we did not examine participants without stereopsis such as strabismus patients.

Our study could confirm the hypothesis of Fergo et al., who hypothesized that surgeons with good stereo acuity can gain from 3D laparoscopy, whereas stereo blind surgeons would be unduly disadvantaged [18]. They highlighted the importance of further studies to assess the impact of stereo vision on surgical performance.

We conclude that a high level of stereo acuity leads to a higher benefit using a 3D laparoscopic system compared to the use of a 4 K-2D system. In addition, surgical experience and fine motor skills are of great importance beyond stereoscopic vision and could partly compensate for a somewhat poorer stereo acuity.

One limitation of this study is that not all participants of the IDOSP trial completed all stereo tests and orthoptic examination. Only 95% of MS (47/49), 41% of NBC (16/39), and 65% of BC (26/40) finally completed stereo acuity tests. Therefore, this subgroup is slightly different from the IDOSP-Study group. As all statistical tests were applied to this subgroup, we do not expect any bias because of this.

Limitations of this study and the IDOSP trial include that the participants used their own glasses for both the parkour and the stereo tests. The glasses might have been not the best possible correction for their ametropia. Especially in participants with beginning presbyopia, they might have been undercorrected for near distance.

Another possible limitation is that the monitor distance in the parkour, which was 130 cm, did not equal the test distance of the stereo tests, which are designed for a distance of 40 cm. This fact might have led to an underestimation of stereo acuity of participants with undercorrected presbyopia. As the subgroups of NBC and BC had a higher mean age than the subgroup of MS and therefore probably a higher percentage of presbyopia, this fact could bias the results. Especially the finding that untrained participants are more dependent on their stereo acuity and benefit more from a 3D monitor system than trained surgeons can partly be explained by the test modality of stereo vision with disadvantages for participants with presbyopia without best correction. However, even at a 130 cm screen distance the stereo acuity can be reduced when spectacles for distance vision are worn by participants with presbyopia. This hypothesis is confirmed by the significant correlation between stereo acuity and parkour performance we found in this study.

Furthermore, the number of participants within different stereo subgroups was quite small in some subgroups and was not equal between subgroups. This could also lead to bias.

Stereo acuity cannot be trained in adults. However, stereo acuity is known to be better when wearing best-corrected glasses [20] especially when surgeons have presbyopia. As the 3D monitor is often placed at a different distance from the normal eye-hand distance in open abdominal surgery, it can be necessary to fit glasses especially for laparoscopic surgery for surgeons with presbyopia.

We furthermore conclude that in future clinical studies on laparoscopic surgery using a 3D monitor system it is of critical importance to evaluate the participants’ stereo acuity and make sure that the stereo acuity is comparable between different study groups as heterogeneity in stereo acuity could otherwise lead to a significant bias of the results.

## Data Availability

The datasets generated and/or analyzed during the current study are not publicly available due to the data security concept of the study and the General Data Protection Regulation of the European Union but are available from the corresponding author on reasonable request.
